# Nurse anesthetists’ experiences using smart glasses to monitor patients’ vital signs during anesthesia care: A qualitative study

**DOI:** 10.1371/journal.pone.0250122

**Published:** 2021-04-21

**Authors:** Charlotte Romare, Per Enlöf, Peter Anderberg, Pether Jildenstål, Johan Sanmartin Berglund, Lisa Skär

**Affiliations:** 1 Department of Health, Blekinge Institute of Technology, Karlskrona, Sweden; 2 Intensive Care Unit, Department of Anesthesiology, Region Blekinge, Karlskrona, Sweden; 3 Institute of Health and Care Sciences, University of Gothenburg Sahlgrenska academy, Gothenburg, Sweden; 4 Department of Anesthesiology, Surgery and Intensive Care, Sahlgrenska University Hospital, Gothenburg, Sweden; 5 Department of Health Sciences, University of Skövde, Skövde, Sweden; University of Birmingham, UNITED KINGDOM

## Abstract

**Purpose:**

To describe nurse anesthetists’ experiences using smart glasses to monitor patients’ vital signs during anesthesia care.

**Methods:**

Data was collected through individual semi-structured interviews with seven nurse anesthetists who had used smart glasses, with a customized application for monitoring vital signs, during clinical anesthesia care. Data was analyzed using thematic content analysis.

**Results:**

An overarching theme became evident during analysis; *Facing and embracing responsibility*. Being a nurse anesthetist entails a great responsibility, and the participants demonstrated that they shouldered this responsibility with pride. The theme was divided in two sub-themes. The first of these, *A new way of working*, comprised the categories *Adoption* and *Utility*. This involved incorporating smart glasses into existing routines in order to provide safe anesthesia care. The second sub-theme, *Encountering side effects*, consisted of the categories *Obstacles* and *Personal affect*. This sub-theme concerned the possibility to use smart glasses as intended, as well as the affect on nurse anesthetists as users.

**Conclusion:**

Smart glasses improved access to vital signs and enabled continuous monitoring regardless of location. Continued development and improvement, both in terms of the application software and the hardware, are necessary for smart glasses to meet nurse anesthetists’ needs in clinical practice.

## Introduction

### Anesthesia care

Many people will encounter anesthesia related to surgery or treatment at some point in their lives, as a patient or as a relative. During anesthesia, sedative drugs are provided that cause changes to vital organ systems, such as the circulatory and respiratory systems. The anesthetized patient’s vital organ functions are maintained and carefully monitored by specialized health care professionals (HCPs), including anesthesiologists and nurse anesthetists (NAs), to provide safe anesthesia care [1]. The NAs remains close to the patient during anesthesia and is responsible for surveillance and life support. In Sweden, NAs has completed one year of post-graduate academic training. With the support of anesthesiologists, NAs induces, maintains, and concludes anesthesia [2]. Surveillance includes monitoring the patient’s vital signs (VSs), such as blood pressure, pulse, and oxygen saturation, as these parameters can provide an early indication of issues that are about to arise [3]. This is an important aspect of patient safety in anesthesia care [1], and monitoring has improved over the last decades [4]. Surveillance also includes assessing other sources of information, such as the patient’s facial expression (tense or relaxed), skin colour (flushed or pale), etc. [3]. The NA has several important responsibilities and holds a key role in ensuring safe anesthesia for patients [5].

### Smart glasses

Smart glasses (SG) are worn like regular eyeglasses, and data can be presented in the user’s field of view through a prism. SG can be connected to Wi-Fi and Bluetooth and display e.g. webpages or pictures. Images and video can be captured with their integrated camera. SG can be used for communication by voice and/or video. Users can control SG via voice- or touch commands or physical input such as eye-blink detection [6]. Research has shown that SG is suitable in situations in which timely access to information, mobility, continuous attention, and hands-free interaction are required [7].

### Smart glasses for anesthesia care

SG have been suggested to improve communication and safety in intraoperative care [8], as well as to facilitate the monitoring of VSs. This has previously been tested in simulated settings [9–11] and clinically used by surgeons monitoring VSs [12], as well as in the similar context of intensive care [13]. A scoping review highlights that research concerning the clinical use of SG in complex care environments is limited [14]. SG require a tailored software (application) for each context and purpose [15,16]. HCPs’ requests regarding both quality of use and desired VSs to make SG a tool for clinical monitoring of VSs and a part of surveillance has recently been described [17,18]. The innovation project SUCCCE used i.a. this information to develop a VSs-monitoring application for SG and conducted feasibility tests [19] in anesthesia care. The aim of the present study was to describe nurse anesthetists’ experiences using smart glasses to monitor patients’ vital signs during anesthesia care. After conducting this study, the authors conclude that the aim was reached.

## Methods

### Design

We conducted a qualitative study within the naturalistic paradigm [20] using an inductive approach [21] as suggested for new research areas [22]. Data was collected through individual semi-structured interviews [20]. To meet the aim and describe the participants’ experiences the methodology used for analysis was thematic content analysis [23]. This enable a deeper understanding since the ontological standpoint is that experiences are complex, subjective and context dependent, rather than that there is an objective truth to find. The researchers were close to and interacted with both the participants and the data as the epistemological stance [20]. To increase trustworthiness, we have strived to address both credibility, dependability, conformability, transferability and authenticity, e.g. by using the checklist to improve trustworthiness provided by Elo and colleagues [24]. The process is described below. The checklist *Standards for Reporting Qualitative Research* (SRQR) [25] was used to further increase the transparency of this study.

### Context

The feasibility tests of SG were conducted in a anesthesia department of a university hospital in Sweden that performs neurosurgical-, hand-, and reconstructive surgery, as well as surgical interventions on ear, nose, and throat. Among those employed at the unit at the time of the study there were 22 NAs (12 female, and 10 male). Two stationary monitors (Philips IntelliVue) in each operating room (OR) allow the NA to monitor VSs—including e.g. blood pressure, oxygen saturation, pulse, and ECG—during surgery. Vital information about the patient’s status, mainly focusing on sedation and ventilation, is also provided by the anesthesia station.

### Participants

Registration of interest to participate in the test group for SG was gathered in connection with an earlier study [18]. Besides that, all HCPs employed at the unit who were interested in participating in the test group could report their interest to the second author (PE) who works as a NA at the unit in which the feasibility tests were conducted. Everyone interested were included in the test group and they were all invited to individual interviews following the feasibility tests. PE managed all contacts regarding SG-related technology, and the project SUCCCE. He also assisted with practical matters, and contextual anesthesiology knowledge during the study.

A total of twelve participants joined the test group—three anesthesiologists and nine NAs. All three anesthesiologists left the unit during the feasibility tests for reasons of further educational pursuits and/or new employment. Hence, only NAs were included in the interviews. Seven of the NAs agreed to be interviewed for this study; see Table 1.

**Table 1:** Participant characteristics.

Variable	Value
Total number of participants	Number = 7
Gender	3 female, 4 male
Age	31–48 years (mean 37 years)
Experience as registered nurse	6–28 years (mean 13 years)
Experience as nurse anesthetist	1–25 (mean 6 years)
Wear prescription eyeglasses	Number = 3

### Technical setup

**Fig 1:**
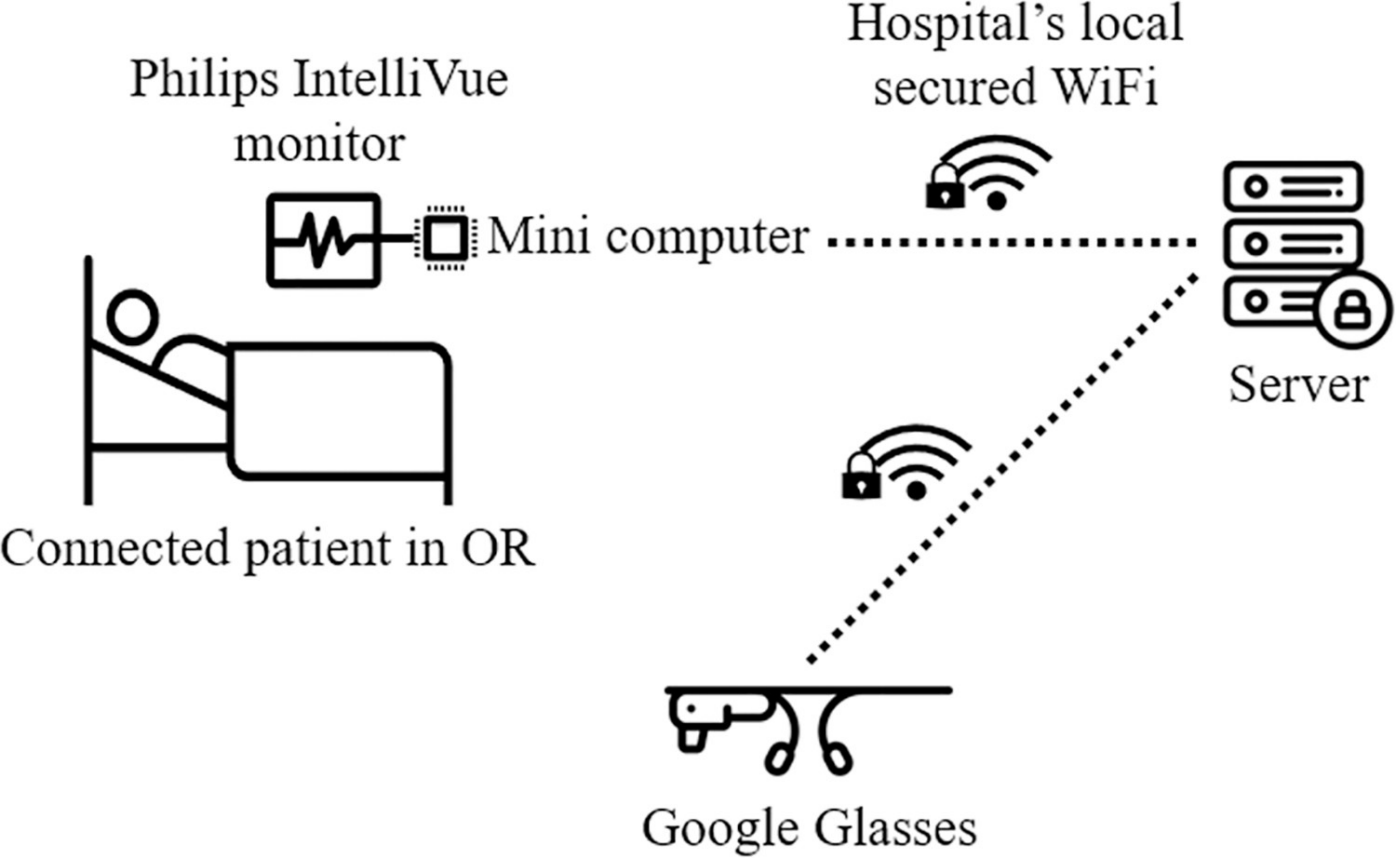
Overview of technical setup. (Some icons made by Freepik from www.flaticon.com).

The patients were connected to Philips IntelliVue surveillance equipment as usual. A mini-computer connected to the IntelliVue device by a serial port extracted, translated, and transmitted the data needed for the application used in the SG. Using the hospital’s secured Wi-Fi where patient data is usually sent, data was sent for queuing and extra processing to a hospital-based middle-ware server. The data was then sent to SG using the same network, see Fig 1.

The SG used as platform for the application in the feasibility tests were Google Glass Enterprise Edition, see Table 2.

**Table 2:** Technical specifications for SG used in this study.

Item	Feature
Processor	Intel Atom, 32 bit
RAM	2 GB
Flash memory	32 GB
OS	Android 4.X
Display	640 x 360 px
Sensors	Ambient light sensor, a digital compass, a wink sensor, a blink sensor, a barometer, a capacitive head sensor (in place of the proximity sensor), a hinge sensor (for determining whether the hinge is open or closed) and assisted GPS & GLONASS.
Communication	Wi-Fi, dual-band 2.4 + 5 GHz 802.11a/b/g/n/ac. Bluetooth LE and HID; supports multiple Bluetooth connections at once.
Camera	5 MP stills and 720p video
Battery	780 mAh
Controls input	Voice, touchpad
Weight	36 g

**Fig 2:**
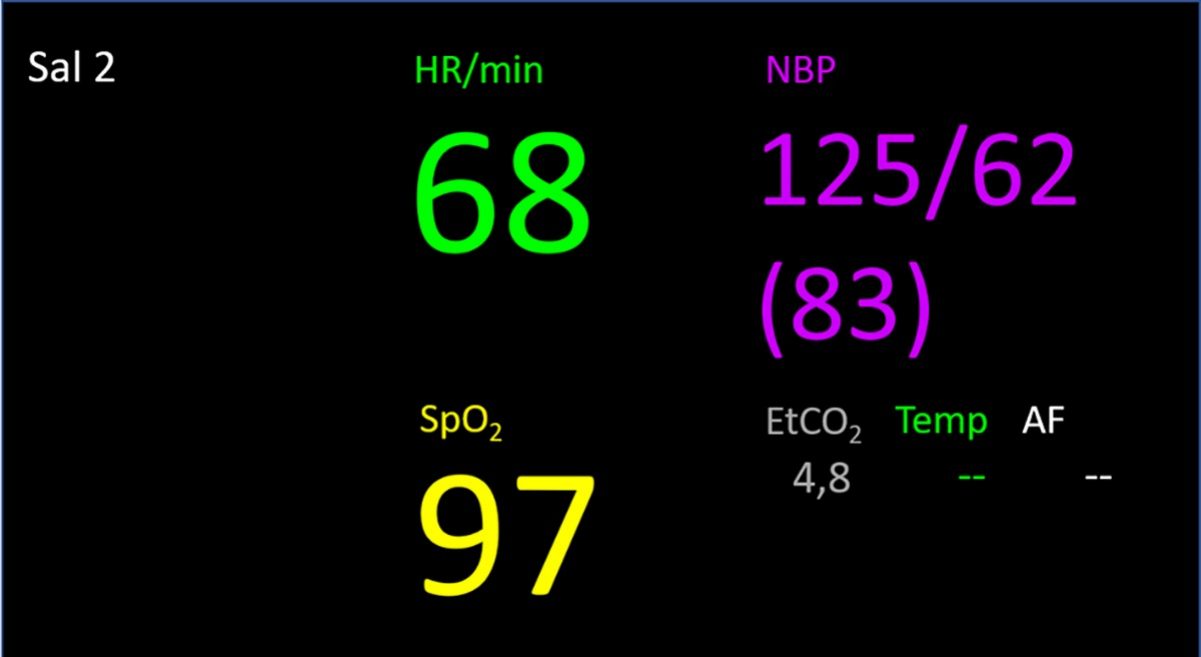
Example view of vital signs in smart glasses. Heart rate (HR) in green; non-invasive blood pressure (NBP) in purple (invasive blood pressure would have been presented in red); oxygen saturation (SpO_2_) in yellow; and end tidal carbon dioxide (EtCO_2_) in grey. Temperature (Temp) and breathing frequency (AF) are not shown in this application version. Sal 2 indicates the current operating room.

**Fig 3:**
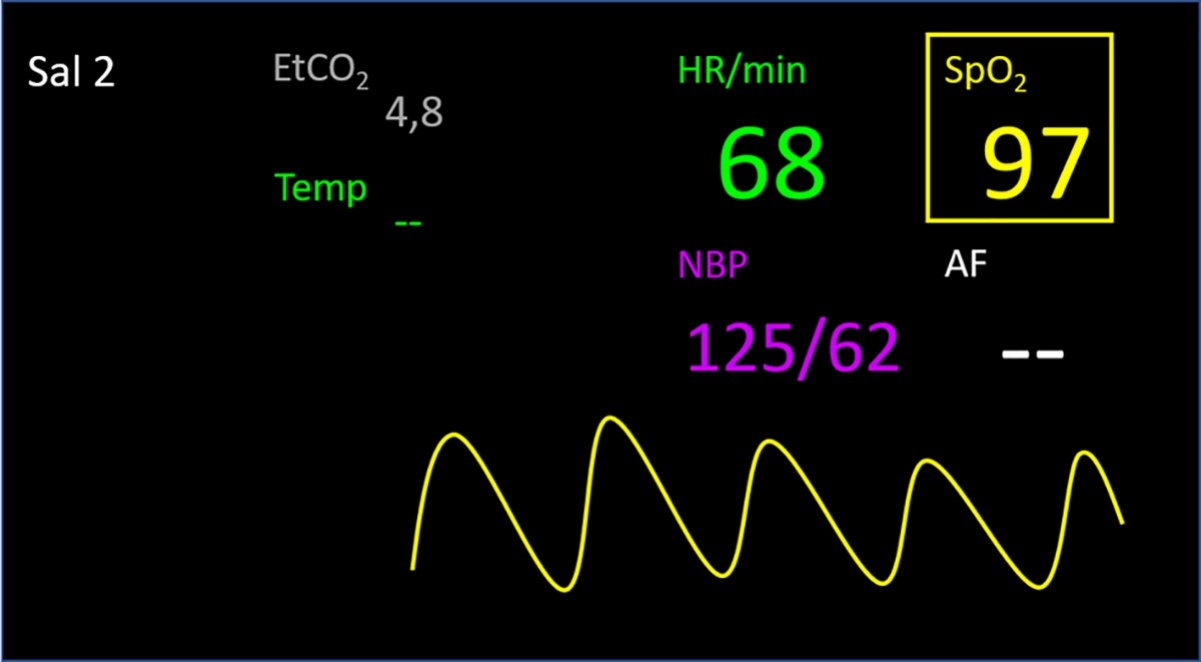
Example view of vital signs and associated oxygen saturation curve (yellow) in smart glasses. End tidal carbon dioxide (EtCO_2_) in grey; heart rate (HR) in green; oxygen saturation (SpO_2_) in yellow (the square around it indicates that the parameter is also being presented by an associated curve); and non-invasive blood pressure (NBP) in purple (invasive blood pressure would have been presented in red). Temperature (Temp) and breathing frequency (AF) are not shown in this application version. Sal 2 indicates the current operating room.

SG was only used to present VSs in the feasibility tests of SG with the tailored application. The tailored application provided ECG, pulse, blood pressure (invasive or non-invasive), oxygen saturation, and end tidal carbon dioxide. VSs were presented numerically, and/or as an associated curve, as requested by HCPs when their views of SG prior to clinical use were described [18]. Participants could decide whether VSs should be presented only numerically, or with numbers and an associated curve. If only numbers were presented, the associated curve could be accessed by choosing the current number in the SG. Only one curve could be presented at a time; see Figs 2 and 3. Alarm limits did not diverge from those set on the stationary monitor. If a VS violated alarm limits, an auditory alarm sounded in SG, as well as from the stationary monitors. The SG used during the feasibility tests could be controlled by voice and via the temple touchpad.

All participants were given a brief introduction on how to use the SG by a member of the SUCCCE project or by PE. They were told to start using the SG in easier, less complex situations and instructed to remove the SG if they felt that using them could entail any risk to patient safety. Before entering the OR, the SG were used to scan a QR code outside the room, to present VSs connected to that specific room. The OR number was displayed in the SG to ensure that VSs from the correct room were being presented. The alarms on the stationary monitors providing VSs in the OR could not be muted through SG during the feasibility tests. The command “mute alarm” only muted alarms in SG; this was out of concern for patient safety, as the rest of the operating team are accustomed to be notified about changes in VSs from alarms on stationary monitors. The participants estimated that they used SG between 10–20 times each (mean number of total uses was 108).

### Ethical approval

Ethical approval for this study was obtained from the Regional Ethical Board in Lund, Sweden (Dnr 2016/773 and 2018/107). The anesthesia department management gave permission for the study to be conducted. Both written and oral informed consent were collected from all participants before the interviews, and they were informed that they could withdraw at any time with no further explanation. All work related to this study was carried out in accordance with the Declaration of Helsinki [26].

### Data collection

Data was collected in May 2019 by CR. CR is a critical care nurse with knowledge of the work performed by NAs’. She was employed at another hospital and had no relationship to the participants in this study. Semi-structured interviews were conducted [20], using a short survey and an interview guide, see S1 Text. In the survey, participants highlighted, among other things, different situations in which they had used SG. Guided by their answers, questions from the interview guide were asked, for example:

○ Tell me about when you used the SG to. . . [participants survey answer].
○ Tell me if there were any positive aspects to this use.
○ Tell me if there were any negative aspects to this use.
○ How would you rate patient safety in this situation, using SG compared to not using SG?

Follow-up questions were asked, such as: Can you tell me more about that, or Can you give an example. Interviews were conducted during working hours in offices within or close to the unit in which the participants worked. All interviews were recorded and transcribed verbatim by CR. Notes were taken during interviews regarding e.g. body language and tone of voice and added to the transcripts. Post-interview procedures were conducted after the first two interviews as suggested [20,24], to improve subsequent interviews. To allow a thorough evaluation of the interview guide, the transcripts were read, and interviews listened to, by CR, PA, JSB and LS. No changes were made to the content of questions; adjustments were made only to the formulation of the questions. This can be seen as pilot testing of the interview guide. All interviews were included in the analysis.

### Analysis

Data was analyzed using thematic content analysis. Initially the transcripts were read several times to become immersed in the data. The process of decontextualization included marking meaning units (words, sentence, or sentences), condensation of meaning units (if needed) whilst still retaining the core, and then labelling them with codes [23]. The marked meaning units were continuously numbered, and notes were added about the situation to which the participants referred. Decontextualization was carried out by CR. During the process of recontextualization, codes were sorted into sub-categories, and sub-categories into categories. The process was not one-way but went back and forth between the different steps [23]. Recontextualization was done by CR, PA, JSB and LS. An audit trail with examples of codes is available in S1 Table. To gain trustworthiness one researcher (CR) was responsible for analysis, and other researchers closely followed up the process, as suggested [24]. Until this point, the analysis was on a manifest level, close to the text. During the process of analysis the latent content became evident, and through interpretation [21] a theme and two sub-themes were created.

## Results

Four categories with sub-categories were created during the thematic content analysis. Two sub-themes became evident, as did an overarching theme, see Table 3.

**Table 3:** Overview of results.

Theme: Facing and embracing responsibility			
Sub-theme: A new way of working		Sub-theme: Encountering side effects	
Adoption	Utility	Obstacles	Personal affect
To become accustomed to SG	To use SG in specific situations	To navigate SG	To be physically affected by SG
To be able to recognize VSs in SG	To manage alarms with SG	To access and assess information provided by SG	To feel uneasy during use of SG
	To maintain control in the situation by SG	To encounter technical issues with SG	
	To use SG while cooperating with others	To identify potential risks with SG	
	To see future potential for SG		

During analysis it became obvious that being a NA entails a great responsibility, and the participants shouldered this responsibility with pride. When working with SG, the responsibility to the patient was given the highest priority, and the NAs tried to use SG in a way that could facilitate their work to provide safe anesthesia care for patients. ***Facing and embracing responsibility*** thus represents the latent content, the theme. This included finding *a new way of working*, incorporating SG into existing routines. It also included *encountering side effects* that could affect both NAs as users, as well as the possibility to use SG as intended.

### A new way of working

SG were a new tool for the participants, and analysis reveals in this sub-theme how NAs strived to incorporate SG in their daily work providing safe anesthesia care for patients.

#### Adoption

The analysis showed that NAs saw SG as a complement to existing monitoring. They needed **to become accustomed to SG,** and there was a learning curve associated with the use of SG. The NAs could use voice and/or touch to control SG; this was included in the learning process. Touch control was found most intuitive to use:

*We’re accustomed to using our fingers to control technology; it feels safe* (NA5).

NAs were so used to working with stationary monitors to monitor VSs that an active effort was required to feel comfortable using SG and to take advantage of its benefits. This worked out well sometimes, but if something divergent happened during anesthesia care, the NAs automatically used the stationary monitors as usual. Because of the possibility to continue using the stationary monitors, the NAs did not feel that the SG posed any risk to patient safety. As SG use became more habitual, they were seen as a potentially helpful tool during anesthesia care, and their adoption in a new way of working was seen as a possibility.

Analysis revealed that it was important **to be able to recognize VSs in SG** for them to be adopted. VSs were presented using the same colors as on the stationary monitors, which was appreciated. The layout was also similar, but not the same. The NAs could choose, according to personal preferences, for VSs to be displayed numerically or by numbers and one associated curve. Curves was considered important to be able to evaluate if the presented number was accurate, to make the new way of working safe.

#### Utility

Analysis identifies NAs’ **use of SG in specific situations**. SG were utilized during induction and intubation. By glancing upwards during intubation, NAs could see for example oxygen saturation; this was seen as positive, and an asset for patient safety:

*They [SG] didn’t get in my way; they don’t disturb my vision*, *and they provide the information I want* (NA6).

According to one NA, moving one’s head to observe VSs on the stationary monitor could cause the position of the hand to shift during intubation, and this could be avoided using SG. The new way of working—utilizing SG to monitor VSs—was considered helpful, but it could not replace collegial support and assistance during demanding procedures such as intubation. SG provided faster access to VSs, as well as access to VSs while performing other tasks (e.g. documentation, working under sterile covers, moving around in the OR), or when NAs had to turn their back on the stationary monitors. Being able to monitor VSs at all times increased the feeling of security. In such situations, the NAs unanimously agreed that SG were an aid.

*But perhaps above all they [SG] are most valuable when one’s back is turned to the monitor*, *etc*., *when one can’t access the stationary monitors right then* (NA3).

When preparing drugs, the NAs had to turn their backs on the patient and the stationary monitors. SG use was seen as positive in such situations, making it possible to maintain focus on the drug preparation. While the utility of SG was seen as less pronounced in smaller ORs, they were not seen to decrease patient safety; patient safety was seen as unchanged or slightly increased also in small ORs.

The analysis revealed that **alarm management with SG** was another asset of the new way of working. NAs appreciated that they were notified through the SG if the VSs were outside accepted values, and that they could quickly evaluate the cause of the alarm, especially when the stationary monitors were out of sight. NAs also experienced that they noticed alarms faster using SG, and that their focus on ongoing tasks could be maintained while managing alarms.

The analysis showed that making it possible **to maintain control in a situation via SG** was an important feature of utilizing SG. NAs could focus on what they were doing and still continue monitoring VSs:

*One feels that one can actually focus fully on one task*, *whilst still feeling the security of knowing what is going on with the patient* (NA5).

This alleviated the NAs’ feeling of stress. SG facilitated monitoring of VSs, and this new way of working made NAs feel that they could maintain control in different situations.

The analysis highlighted aspects of **using SG while cooperating with others**, both patients and colleagues. Only a few of the NAs had utilized SG during encounters with awake patients. While some of those patients had noticed the SG, and asked questions out of curiosity, most patients did not comment on the SG at all. NAs who had used SG during encounters with awake patients did not feel that the relation was affected in a negative way, rather the opposite. When using SG, the NA could focus on the patient and did not need to look away to watch stationary monitors; this was seen as potentially increasing the patient’s feeling of security:

…*you could concentrate on standing just behind the patient [during induction]*, *and the patient could look up and see me*, *and I could look at the patient instead of turning my head back and forth to look at the monitors* (NA6).

Some NAs had used SG when teaching students and introducing new colleagues. When the student was ready to be left alone with the patient, NAs felt that SG made it possible to keep an eye of the patient and react from outside the room if needed. Using SG as a complement during tutoring was seen as an improvement to patient safety. One NA had used SG in cooperation with a colleague who was outside the OR to monitor the VSs of an instable patient. When the patient had an arrythmia, they could both watch the ECG and discuss it:

*We found it very clever*, *it was a great way to put them [SG] to use /*. . .*/ we were both positive* (NA3).

The new way of working—utilizing SG to cooperate with others—was considered positive, and colleagues had shown positive interest in SG. However, being bedside and close to the patient cannot be replaced by monitoring VSs through SG.

The analysis showed that the NAs **saw future potential for SG**, and suggestions were made, e.g.: use at other locations such as the intervention room and larger ORs, during x-ray and computer tomography, during transports, during intensive care, when working with multiple patients, and use of SG cameras during intubation and tutoring. Some of the NAs suggested further testing and evaluation of some situations that had already been tested: intubation, tutoring, and cooperation with colleagues (both other NAs and anesthesiologists) at a distance. The possibility to mute alarms on the stationary monitors through SG was seen as a valuable future benefit; being able to quickly mute alarms in the room through SG was seen as possibly reducing stress from noise for patients. Such utilization of SG was seen to hold potential to improve anesthesia care.

### Encountering side effects

During analysis it became clear that new technology is not integrated in anesthesia care effortlessly, even if the participants stated that they were skilled technology users and accustomed to high-tech environments. This sub-theme reveals that, despite the efforts made, SG could be challenging to use during anesthesia care.

#### Obstacles

Voice control was one way **to navigate SG**, and the analysis showed that all NAs experienced problems with this feature. Controlling SG by voice was difficult, regardless of whether the environment was quiet or loud, and voice commands had to be repeated multiple times. NAs tried different levels of tone, different pronunciation, standing in a corner to give verbal commands to SG, or even going outside the OR to successfully command their SG. Furthermore, SG had also reacted to the voice of other team members in the OR. This resulted in frustration and uncertainty:

*I don’t know what I’m doing wrong*… *They [SG] just don’t work for me*, *that’s just how it is*… *I have tried every possible ways of talking to those glasses* (NA4).

One side-effect of talking to SG and repeating commands were concerns about disturbing others in the OR; this demonstrates that NAs show respect and responsibility towards others. Wrong menus were sometimes entered inadvertently, and the menu structure was found to be non-intuitive; navigating SG was thus an obstacle.

The analysis revealed that **accessing and assessing information provided by SG** was also an obstacle. In order to see VSs in SG, NAs had to glance upwards and to the right, an active action that was experienced as tiring. It could be difficult for the eye to focus on VSs provided in SG, and shifting between information in SG and in the surroundings was perceived as hard. But when NAs had found the correct focus, information in SG was clearly visible. The curves provided were perceived as slightly different from those on the stationary monitors. NAs stated that it was possible to notice arrythmias, but not to interpret details in curves provided in SG. These obstacles were side effects not noticed while working with stationary monitors.

The analysis found that NAs **encountered technical issues with SG**. The NAs thought that patient safety could increase when SG worked as intended, but they had all experienced side effects in form of technical issues. Technical issues included frequent system shut-downs, switching menus, and returns to the start menu:

*Then there were also issues with the SG suddenly turning off*. . . *especially when an alarm went off*, *and then they just shut down*. . . *and there’s not a single number*! (NA3).

Frequent restarts were also perceived as negatively affecting battery life. Battery life was seen as a limitation for clinical use of SG, and it was common that the SG battery ran out before the surgery ended. Some NAs also experienced SG connectivity as an obstacle, observing that SG dis-connected and re-connected again. Slight delays in the presentation of VSs in SG were noticed, as well as some discrepancy for VSs (mainly regarding the invasive blood pressure), compared to the parameters shown on stationary monitors. For arrythmias even a slight delay was seen as a negative aspect, since these events need immediate attention, and this could cause decreased trust in SG. Some NAs saw VSs from another OR presented, possibly VSs from the OR in which the SG had been used the previous day. NAs reacted differently to the obstacles caused by technical side effects; some said that it did not affect them, and some felt frustration and irritation. The reactions differed from day to day and depended on the level of stress in the situation. The NAs experienced that focus could move from the patient to SG when technical issues occurred, and that this could affect patient safety negatively; in response, they removed the SG, demonstrating their responsibility towards the patient:

*Trying to restart them [SG] and figure out what went wrong drew focus away from the patient /*. . .*/then they became more of a problem than an aid* (NA1).

Losing focus on the patient was one of the **identified potential risks with SG** that was found as a side effect in the analysis:

*SG mustn’t take the focus from the surgery*, *that’s how I feel* (NA1).

According to NAs, when unaccustomed to something (such as SG), it is easier to become distracted and for focus to be diverted. Other risks identified were muting alarms without having all the information (for example from curves), or that alarms might inadvertently be muted from outside the OR, making the NA in the OR miss events (this was not possible during these feasibility tests). Reducing the number of colleagues in the OR because it was possible to get assistance from a distance was not seen as an option, but rather a potential patient safety risk. Analysis also revealed that NAs who had worn SG with awake patients reflected on whether the patients had thought and wondered about SG, but perhaps did not dare to ask the NA about them. Reflecting on potential risks of various nature is one way of facing and embracing responsibility for safe patient care.

#### Personal affect

The analysis disclosed that NAs were **physically affected by SG,** as another type of side effect. For NAs who wore prescription glasses, the experience of combining these with SG differed. Some were able to wear both, but for others this was impossible. The prescription glasses had to be removed in some cases, which was seen as infeasible during clinical work. SG were found heavy and NAs reported that they pressed against the temples and caused pain from the ears. Some NAs encountered side effects in terms of headaches and feelings of tiredness (in both the head and the eyes) that were seen as related to gazing upwards to access VSs in SG.

During the analysis it became evident that SG users could **feel uneasy during use of SG**, as an emotional side effect. They were still willing to use SG if it could improve anesthesia care, hence the responsibility towards the patients is evident. Speaking to SG made some feel foolish, and colleagues had reacted to NAs talking to SG—not with complaints but with sarcasm and teasing. SG also affected NAs physical appearance, both when wearing both pairs of glasses (which could make one feel foolish), but also otherwise:

*You look ridiculous in them*, *but what can you do*. . . (NA4).

Some NAs did not feel comfortable using SG during encounters with awake patients and chose to start using SG when the patient had been anesthetized:

*I haven’t dared to try [using SG during an encounter with an awake patient] /*. . .*/ they might start thinking about what they [SG] might be instead*. . . *But maybe that’s just a stupid thing*. . . *maybe*. . . (NA2).

The analysis indicated that using SG with awake patients would feel better when more accustomed to and comfortable with SG use during anesthesia care.

## Discussion

### Discussion of results

The results reveal that SG affected more than just monitoring VSs; they affected most aspects of anesthesia care, as well as the users. NAs’ responsibility, and how they face and embrace it while caring for patients, is demonstrated by their reflective and thoughtful approach. The NAs in this study strived to establish a new way of working in which SG were integrated into existing routines to provide safe anesthesia care. It took some time for NAs to adopt SG in clinical practice, but then they found utilization employing SG for several purposes, for example accessing VSs and alarms regardless of location. The results also show that NAs encountered problems along the way, and obstacles made clinical use difficult from time to time due to e.g. technical issues. The results highlight how NAs use SG with care and a reflective approach, hence they face and embrace responsibility for the patients, and for providing safe anesthesia care.

The results show that SG increased access to VSs during anesthesia care, regardless of the NA’s location. This has been shown earlier in similar [13,27,28], and simulated [29,30] settings, and is now accordingly also experienced by NAs during clinical anesthesia care. Information provided in the users’ field of view can improve response time and increase attentiveness [31] as well as situation awareness [12,27]. Providing VSs in SG has previously been found to enable increased and maintained focus on ongoing tasks [12,30], and the results of this study supports this. Being given access to all information allow HCPs to make better decisions, for example if an ongoing task needs to be interrupted in favor of a more important event [30]. This is seen as an asset for improved quality of care and improved patient safety [13], and is applicable to anesthesia care as well.

The results provide information about technical issues that NAs encountered using SG. Connectivity, frequent system shut-downs and restarts, and battery life caused irritation. This was also an area about which anesthesia HCPs were apprehensive before clinical use [18]. The results do not provide information about whether the technical problems NAs encountered in this study were caused by the Wi-Fi, the customized application, or by the SG as platform, or if they were a combination of the aforementioned reasons. These issues, and the challenge of locating the source of the problem, have been highlighted earlier [32]. Regardless of the origin of the technical issues, research has shown that there is a high risk of distraction when technology fails [33,34] moving focus from the patient to technology, both during education [34] and in intensive care [35]. The results from this study show that this is also true during anesthesia care. The participants in this study paid attention to their reactions while using SG and removed the SG if they felt distracted. This is one of many examples in which the theme is obvious, and NAs clearly show how they face and embrace their responsibility for the patient and providing safe anesthesia care. This is in line with earlier research findings [36–39].

In this study, some patients questioned and commented on SG to the NAs out of curiosity, as also noted in earlier research [28,40]. A few studies with quantitative design have reported on patients’ perceptions of SG used in their care in a hospital setting [41–43], but qualitative studies about patients’ perceptions of SG in an anesthesia context seem to be missing. The patients’ views of SG were something on which NAs in this study reflected, and here too NAs demonstrate how they face and embrace responsibility, doing (and using) what is best for the patients to make them feel secure. While investigating patients’ perceptions further was beyond the scope of this study, this is an interesting and important topic to address in future research. Data security and privacy are ethical considerations often mentioned in relation to SG use in health care [15,44,45]. Since these are known potential problems, they were addressed in the development of the application used in these feasibility tests. But as the results from this study show, there are other ethical aspects to using SG; it has for example been stated that SG affects social interaction [45–47]. Considering how SG will affect nurse-patient interaction during anesthesia care is interesting and important, since the nurse-patient relationship has a crucial role for patient safety [48].

The results of this study reveal that alarms were noticed faster when using SG. This finding is supported by others [27,30], and the possibility to improve alarm management with SG is endorsed for future development. This feature was highlighted before clinical use in both an anesthesia- [18] and intensive care context [17]. A review of physiological monitor alarms found that between 74–99% of them were non-actionable, and an increasing number of alarms was associated with longer response time from nurses [49]. That review did not include studies from an anesthesia context, but it is known that alarms can distract anesthesiologists during demanding procedures [27]. An excessive amount of nuisance alarms can lead to alarm fatigue [50,51], i.e. desensitization by too many false or non-actionable alarms [51]. Alarm management and alarm fatigue have been on the Emergency Care Research Institute’s (ECRI’s) annual list of technology hazards in health care for years now, and they are still on the list in 2020 [52]. Alarm management is clearly a patient safety issue requiring attention, and an interdisciplinary approach is suggested to address the topic [51]. It has also been discussed that a user-centered design is vital to make wearable technology useful in ORs and to protect patient safety [53]. This study can be part of that, providing end-users’ experiences.

Technology is known to both improve and impair patient safety [54]. Continued technology research with a nursing focus [34] and field-based research [54], such as this study, is essential to create evidence to provide safe care for patients [34,54], and more research is needed regarding SG in anesthesia care.

### Methodological considerations

Several aspects of trustworthiness are met through the fact that this study is a result of cooperation and agreement among co-researchers as suggested [23,24]. The interviews were conducted by CR. The two first interviews were thoroughly evaluated together with PA, JSB, and LS who have extensive interviewing experience. Only minor changes were made after the first two interviews, indicating that performance was sufficient. These are aspects related to dependability of this study. The number of participants can be considered small, but if the research question is narrow and the topic specific, as in the study at hand, a smaller number of participants can suffice to reach data saturation [20]. Even if the participants were few, the number of uses of SG was high (total mean of 108 uses), which is a strength of this study. The process of analysis was continuously discussed by CR, PA, JSB, and LS to increase credibility [20,23]. We strove to carefully describe and report our work to enable others to follow the process [20,21], using SRQR [25]. The final results were read by PE and PJ, both of whom have contextual knowledge about anesthesia care; furthermore PE has experience using SG in this context [55]. This provided valuable confirmability [23]. These are aspects related to credibility of the study. After conducting the data collection and analysis, it was concluded that the data was rich and generated new knowledge within this new area of SG use.

The participants included in this study stated that they were experienced technology users, both in their profession, where they worked in a high-tech environment, and in their everyday lives. They also stated that they were interested in trying new technology, mainly at work. Being interested in innovations might indicate that the participants are so-called “early adopters” [56] and that the results from this study might not be representative of others. However, it has been suggested that critique from early adopters is more profound than the critique of those who were initially skeptical [56]; this can be seen as a strength of this study.

The findings are connected to this specific context and setting, as are the conclusions. However, the findings can be valuable for other contexts and settings as well. Generalization and transferability are left to the reader’s discretion.

## Conclusions

In conclusion, SG with a customized application improves access to VSs and enables continuous monitoring regardless of location. The application used during these feasibility tests was customized to this specific ward and can not necessarily be used in other settings and contexts without modifications. The results from this study show that there is potential for SG in anesthesia care, but improvements are necessary. Based on the results, continued development and improvement are suggested—both of the application for monitoring VSs, and for SG hardware, to meet NAs’ needs in anesthesia care. Meanwhile, further testing in simulated setting is proposed.

## Supporting information

S1 Table Audit trail for process of analysis. The table provides examples of codes for each sub-category.
(PDF)

S1 Text Interview guide. The interview guide is provided in both original language (Swedish) and translated into English.
(PDF)
