# Supplementary material for: Nurse anesthetists’ experiences using smart glasses to monitor patients’ vital signs during anesthesia care: A qualitative study
Source: PLoS One. 2021 Apr 21;16(4):e0250122. doi: 10.1371/journal.pone.0250122 (PMC8059852; doi:10.1371/journal.pone.0250122)
Supplement: S1 Table — The table provides examples of codes for each sub-category. (PDF) [file pone.0250122.s001.pdf]

S2 Table: Audit trail for process of analysis

Theme	Facing and embracing responsibility												
Sub-themes	A new way of working							Encountering side effects					
Categories	Adoption		Utility					Obstacles				Personal affect	
Sub-categories	To become accustomed to SG	To be able to recognize VSs in SG	To use SG in specific situations	To manage alarms with SG	To maintain control in the situation by SG	To use SG while cooperating with others	To see future potential for SG	To navigate SG	To access and assess information provided by SG	To encounter technical issues with SG	To identify potential risks with SG	To be physically affected by SG	To feel uneasy during use of SG
3 examples of codes for each sub-category	Use as a complement<br><br>Learning curve<br><br>Start of easy	Colours<br><br>Layout<br><br>Curves	Intubation<br><br>Preparing drugs<br><br>Can't see wall-mounted monitors	Get alerts<br><br>Mute alarms<br><br>View alarms	Increased focus on task<br><br>Important vital signs<br><br>Keep track	Students<br><br>With patients<br><br>Analyse events together	Handle alarms<br><br>Possible areas of use<br><br>Continue testing	Repeat commands<br><br>Don't want to interrupt<br><br>Difficult menu structure	Analysing curves<br><br>Get focus in the prism<br><br>Gazing upwards	Delay<br><br>Battery life<br><br>Switching menus	Focus on wrong things<br><br>Not replace human presence<br><br>Alarm management	Headache<br><br>Own prescription glasses<br><br>Pain from ears	Feeling stupid<br><br>Reactions from others<br><br>Looking stupid
