# Supplementary material for: Nurse anesthetists’ experiences using smart glasses to monitor patients’ vital signs during anesthesia care: A qualitative study
Source: PLoS One. 2021 Apr 21;16(4):e0250122. doi: 10.1371/journal.pone.0250122 (PMC8059852; doi:10.1371/journal.pone.0250122)
Supplement: S1 Text — The interview guide is provided in both original language (Swedish) and translated into English. (PDF) [file pone.0250122.s002.pdf]

S1 Text: Interview guide

Original interview guide in Swedish

Kort presentation av mig och av projektet.

Syftet med studien är att beskriva anestesisjuksköterskors erfarenhet av att använda smarta glasögon inom anestesisjukvård

Frågor från deltagaren innan inspelning startar?
Verifierar samtycke.

Bakgrundsuppgifter:

- Kön.
- Hur gammal är du?
- Antal år som sjuksköterska?
- Antal år som anestesisjuksköterska?
- Bär du egna glasögon?

- Ungefär hur många gånger har du använt de smarta glasögonen i ditt dagliga arbete?
- Är du intresserad av nya tekniska produkter?
- Om du skulle gradera din vana av teknikanvändning på en skala från 0-10, där 0 är helt ovan och 10 är maximal vana. Vad skulle du sätta för siffra då?

Underlag för vidare intervju utgår från fråga 8-10 i ett logg-blad.

8. I vilka situationer har du använt smarta glasögon idag?

☐ På op-sal under pågående operation, från första uppkoppling till avfärd mot postop
☐ På op-sal under pågående operation, EJ under induktion
☐ Tysta larm
☐ Vid intubation
☐ Larmansvarig anestesisjuksköterska
☐ CVK-inläggning
☐ Varit PAL på olika platser
☐ Som handledare åt student/kollega
☐ Övervaka vitalparametrar utanför op-sal
☐ Annat:........................

9. Vad var positivt med användningen?

☐ Underlättade mitt arbete
☐ Minskat känslan av stress
☐ Förenklad kommunikation
☐ Ökad känsla av trygghet
☐ Ökad känsla av kontroll
☐ Bättre ljudmiljö
☐ Vet inte
☐ Annat:........................

10. vad var negativt med användningen?

☐ Svårt att se parametrarna
☐ Komforten
☐ Handhavandet
☐ Röststyrningen
☐ Touchstyrningen
☐ Batteritiden
☐ Tappade fokus
☐ Ökat känslan av stress
☐ Tekniska problem
☐ Vet inte
☐ Annat:........................

- Berätta vad det är du kan se i glasögonen.
- Berätta hur värdena visas för dig (layout, färger, kurva/siffra).
- Berätta om hur du kan styra eller kontrollera glasögonen.

Ta en situation i taget:

- Berätta om när du använde glasögonen för att .... (situation från logg-blad, fråga 8)
- Berätta om det var något positivt med användningen.
- Berätta om hur glasögonen kunde stödja dig i den situationen.
- Berätta om det var något negativt med användningen.
- Berätta om du noterade någon risk eller problem i den här situationen.
- Berätta hur glasögonen fungerade att använda kliniskt i den här situationen.
- Gradera patientsäkerheten i den här situationen om du jämför med och utan glasögonen. Använd en skala från 0-10.
- Varför blev det just den siffran på skalan?

- Kan du berätta hur patienterna har reagerat på glasögonen?
- Skulle du kunna tänka dig att bära glasögonen framför vakna patienter eller anhöriga?

Exempel på följdfrågor

- Kan du berätta mer om det?
- Hur menar du då?
- Kan du ge ett exempel?

Om det blir fortsatta tester av glasögonen, skulle du då vara intresserad av att fortsätta som testperson?

Har du något som du tillägga innan vi avslutar intervjun?
Tack för att du tog dig tid för att delta!

Interview guide translated into English

Short introduction of me and the project.

The aim of this study is to describe nurse anesthetists' experiences using smart glasses to monitor patients' vital signs during anesthesia care.

Any questions from participant before recording starts?
Verify consent.

Background information

- Gender.
- How old are you?
- Years as nurse?
- Years as nurse anesthetist?
- Do you wear prescription glasses?

- Approximately how many times have you used the smart glasses in clinical practice?
- Are you interested in new technology?
- Suppose you were to grade your experience to use technic equipment using a scale from 0-10, where 0 is the lowest grade and 10 is maximum. What number would you give yourself?

Further interview is based on questions 8-10 in a log sheet.

8. In which situations have you used smart glasses today?

☐ In OR during surgery, from first encounter to departure to PACU
☐ In OR during surgery, NOT during induction
☐ To mute alarms
☐ During intubation
☐ As NA in charge of alarm pager
☐ During insertion of central line
☐ As physician in charge of patients in different locations
☐ As mentor for student or new colleague
☐ To monitor patient vital signs outside OR
☐ Other:.......................

9. What was positive with the use?

☐ Made my work easier
☐ Decreased feeling of stress
☐ Eased communication
☐ Increased sense of security
☐ Increased feeling of control
☐ Improved sound environment
☐ Don't know
☐ Other:.......................

10. What was negative with the use?

☐ Hard to see vital signs
☐ Comfort
☐ Handling
☐ Voice control
☐ Touch control
☐ Battery life
☐ Lost focus
☐ Increased feeling of stress
☐ Technical issues
☐ Don't know
☐ Other:.......................

- Tell me what you can see in the glasses.
- Tell me how this is presented to you (layout, colour, curve/number).
- Tell me how you can control the glasses.

Going through one situation at the time:

- Tell me about when you used the glasses to .... (situation from log sheet, question 8)
- Tell me if there were any positive aspects to this use.
- Tell me about how the glasses could offer you support in this situation.
- Tell me if there were any negative aspects to this use.
- Tell me if you noted any risk or problem in this situation.
- Tell me about how the glasses worked out during clinical use in this situation?
- How would you rate patient safety in this situation, using the glasses compared to not using the glasses? Use a scale from 0-10.
- Motivate your rating.

- Tell me how patients have reacted to the glasses.
- Would you consider wearing the glasses in front of awake patients or their relatives?

Example of follow up questions:

- Can you tell me more about that?
- Can you explain?
- Can you give an example?

If there will be continued testing of the smart glasses in clinical practice, would you be interested in continuing as a participant in the test group?

Is there anything you would like to add before we end the interview?
Thank you for participating!
